# A Novel Terpenoid from *Elephantopus Scaber* – Antibacterial Activity on *Staphylococcus Aureus*: A Substantiate Computational Approach

**Published:** 2008-09

**Authors:** P. Daisy, Salu Mathew, S. Suveena, Nirmala A. Rayan

**Affiliations:** *Bioinformatics Centre (BIF), Department of Biotechnology & Bioinformatics, Holy Cross College, Tepakulam, Tiruchirapalli-620002, India*

**Keywords:** antibacterial, autolysin, terpenoid, CADD, chemsketch, hex, pass prediction

## Abstract

*Staphylococcus aureus* has gained much attention in the last decade as it is a major cause of the Urinary Tract Infection in Diabetic patients. The Extended Spectrum β-Lactamases (ESβL) producers are highly resistant to several conventional antibiotics. This limits the therapeutic options.Hence efforts are now taken to screen few medicinal plants, which are both economic and less toxic. Among the several plants screened, we have chosen the acetone extract of *Elephantopus scaber* from which we purified a new terpenoid for our study. Its structure was generated using CHEMSKETCH software and the activity prediction was done using PASS PREDICTION software. We have confirmed the mechanism of anti-bacterial effect of terpenoid using Computer – Aided Drug Design (CADD) with computational methods to simulate drug – receptor interactions. The Protein-Ligand interaction plays a significant role in the structural based drug designing. In this present study we have taken the Autolysin, the bacteriolytic enzyme, that digest the cell wall peptidoglycon. The autolysin and terpenoid were docked using HEX docking software and the docking score with minimum energy value of -209.54 was calculated. It infers that the terpenoid can inhibit the activity of autolysin by forming a strong atomic interaction with the active site residues. Hence the terpenoid can act as a drug for bacterial infections. Further investigations can be carried out to predict the activity of terpeniod on other targets.

## INTRODUCTION

Diabetes Mellitus (DM) is the most prevalent chronic disease in the world affecting nearly 25% of the population. It is characterized by the elevation of blood sugar level that in turn leads to the excretion of glucose through urine. A higher glucose concentration in urine serves as a culture media for the pathogenic microorganisms as well. The risk of developing infection in DM patients is higher (10, 28) and Urinary Tract (UT) is the most common site for infection (24). *Staphylococcus aureus* (*S.aureus*) is one such organism which multiplies in the UT of DM patients.

Medicinal plants have been used for centuries as remedies for human diseases because they contain components of the therapeutic value. Recently the acceptance of traditional medicine as an alternative form of health care and the development of microbial resistance to the available antibiotics has led authors to investigate the antimicrobial activity of medicinal plants (6, 17, 22).

Drug resistance is one of the most serious global threats to the treatment of infectious diseases (21, 27). Therefore, actions must be taken to reduce this problem, for example, to control the use of antibiotic, and develop research to better understand the basic mechanisms of resistance and to continue studies to develop new drugs, either synthetic or natural. Among the several drug resistant bacteria, β lactamase production is the most important mechanism of resistance to penicillin and cephalosporins. The mechanism of this resistance was the production of Extended Spectrum β- Lactamases (ESBLs) (3). Treatment of infections caused by these resistant bacteria has become very difficult, since they are resistant to many antibiotics. This limits therapeutic options (33). Therefore, alternative methods of treatment are sought after.

There is a continuous and urgent need to discover new antimicrobial compounds with diverse chemical structures and novel mechanisms of action for new and re-emerging infectious diseases. Therefore, researchers are increasingly turning their attention to folk medicine, looking for new leads to develop better drugs against microbial infections (11). Among the several plants screened, *Elephantopus scaber* (*E.scaber*), a member of the family Asteraceae known for its medicinal properties was also reported to posses antimicrobial activity (1). Yet, there were no reports on the role of neither the plant nor its compound against the drug-resistant human pathogens. Hence this plant was chosen. A new terpenoid was isolated from the acetone extract of *E.scaber*. Biological testing of the compound demonstrated a significant anti-bacterial activity against a few multi drug-resistant ESBL producing clinical isolates (20). But the mechanism of the anti-bacterial effect of the compound was not clearly understood to overcome this we tried in nonconventional methods of drug designing by the use of Bioinformatics approaches.

Bioinformatics is seen as an emerging field with the potential to significantly improve how drugs are found, brought to the clinical trials and eventually released to the marketplace. Bioinformatics can be thought of as a central hub that unites several disciplines and methodologies. Computer-Aided Drug Design (CADD) is a specialized discipline that uses computational methods to simulate drug – receptor interactions. CADD methods are heavily dependent on bioinformatics tools, applications and databases. As such, there is considerable overlap in CADD research and Bioinformatics.

Methods developed to facilitate and speedup the drug designing process are Rational Drug Design (RDD). These processes are used in biopharmaceutical industry to discover and develop new drugs. RDD uses a variety of computational methods to identify novel compounds. One of those methods is docking of drug molecules with receptors. The site of drug action, which is ultimately responsible for the pharmaceutical effect, is a receptor.

Docking allows the scientist to virtually screen a database of compounds and predict the strongest binders based on various scoring functions. It explores ways in which two molecules, such as drugs and an enzyme receptor fit together and dock to each other well. The molecules binding to a receptor, inhibit its function, and thus act as drug. Complexes was identified via docking and their relative stabilities were evaluated using molecular dynamics and their binding affinities, using free energy simulations.

Autolysins are bacteriolytic enzymes that digest cell wall peptidoglycan of the bacteria that produce them (32) although potentially lethal; autolysins appear to be universal among bacteria that possess peptidoglycan. Peptidoglycan the substrate of autolysins is a polymer of amylo sugars cross linked by short peptides which forms a covalent matrix that surrounds the cytoplasmic membrane and constitutes the major skeletol component of the cell wall. It is a member of the Metalloprotease Gly-Gly endopeptidase family with PFAM ID PF01551.

The possibility that autolysins are involved in selective removal of peptidoglycan has led to proposals that they are involved in numerous cellular processes including cell growth, cell-wall turnover, peptidoglycan maturation, cell division, separation, motility, chemotaxis, genetic competence, protein secretion, differentiation and pathogenicity (5, 14). Lysis is caused by the presence in the microorganisms of autolytic enzymes (autolysins) which specifically hydrolyse mucopeptide polymers in the bacterial cell wall. The attack occurs, at least in some species, in a very restricted area around the point at which the bacteria will divide (19).

Keeping rational drug designing in mind we made an effort to design natural drug for UT infection caused by *S.aureus*, using a novel terpenoid compound isolated from *E.scaber* by docking against autolysin.

## MATERIALS AND METHODS

The dried and powdered *E.scaber* plant was used to prepare acetone extract which was then subjected to fractionation on silica gel. The most bioactive fraction obtained from acetone extract of *E.scaber* was selected for preliminary phytochemical screening. Test for alkaloids, steroids, flavanoids, Terpenoids and proteins were carried out according to the standard methods (18). A new Terpenoid was purified for the study.

The bacterial isolates were screened for ESBL production following a double disc synergy test (25). The anti-bacterial activity of the Terpenoid was evaluated by the disc diffusion method (4). The Minimal Inhibitory Concentration (MIC) and Minimal Bactericidal Concentration (MBC) were determined for the Terpenoid by broth dilution method (2).

**Figure 1:**
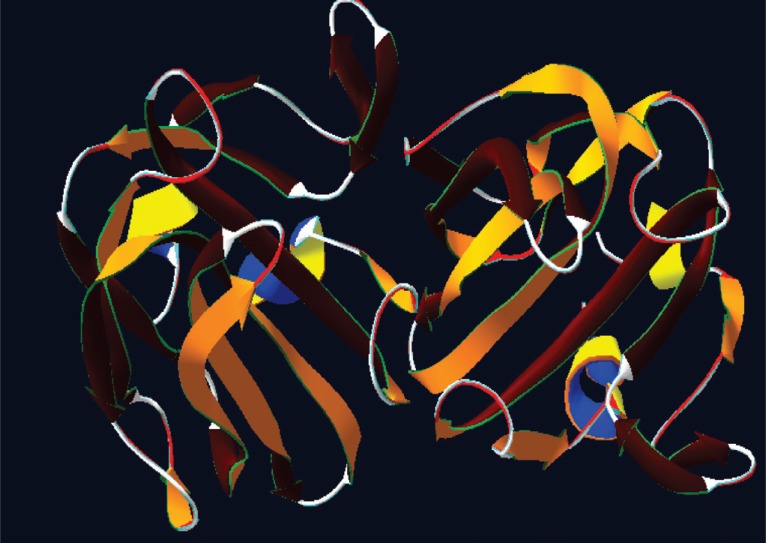
3D structure of the autolysin PDB id 2B0P.

For docking study bioinformatics softwares like Pass Prediction, ChemSketch and Hex docking were used. Based on the literature, the present study was confined to the Terpene family of drugs showing antibacterial activity on membrane protein autolysins. The crystal structure of *S. aureus* LytM (13) with resolution 1.50 A. PDB ID 2B0P retrived from PDB contain chain A and chain B,with PFAM ID PF01551 Peptidase family. Structure contains a long and narrow groove that consists the active site (Fig. 1).

ACD/ChemSketch software is an integrated software package from Advanced Chemistry Development Inc. for drawing chemical structures, 3D optimization algorithm allows the planar (2D) structure from Chem-Sketch to be rapidly translated into a realistic 3-dimensional structure. It is based on the modified molecular mechanics which take into account, bond stretching, angle bending, internal rotation and Van der Waals non-bonded interactions. The 3D optimization algorithm is a proprietary version of molecular mechanics with the force field initially based on CHARMM parameterization (7, 8). CHARMM: A program for macromolecular energy, minimization, and dynamics calculations. By the help of CHEMSKETCH software we have generated the 3D structure of the molecule. The docking analysis of terpenoid with target protein was carried by HEX docking software.

To study the interaction here we have used Swiss pdb viewer. Deep View - Swiss-PdbViewer is an application that provides a user friendly interface allowing analyzing several proteins at the same time. H-bonds, angles and distances between atoms are easy to obtain.

## RESULTS

**Figure 2:**
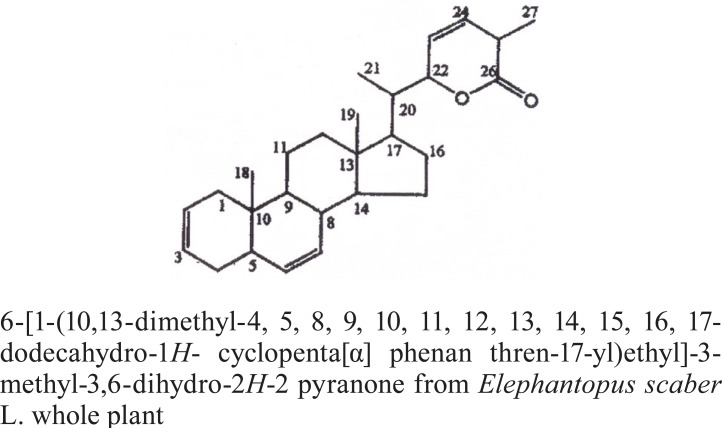
NMR study result of Terpenoid.

**Figure 3:**
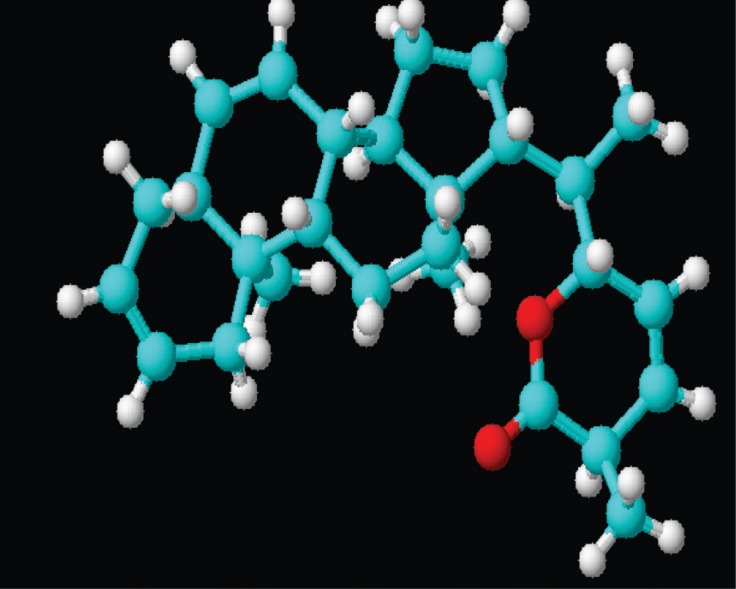
3D structure of the compound Terpenoid (809/CHEM/2007) extracted from *E.Scaber*.

**Figure 4:**
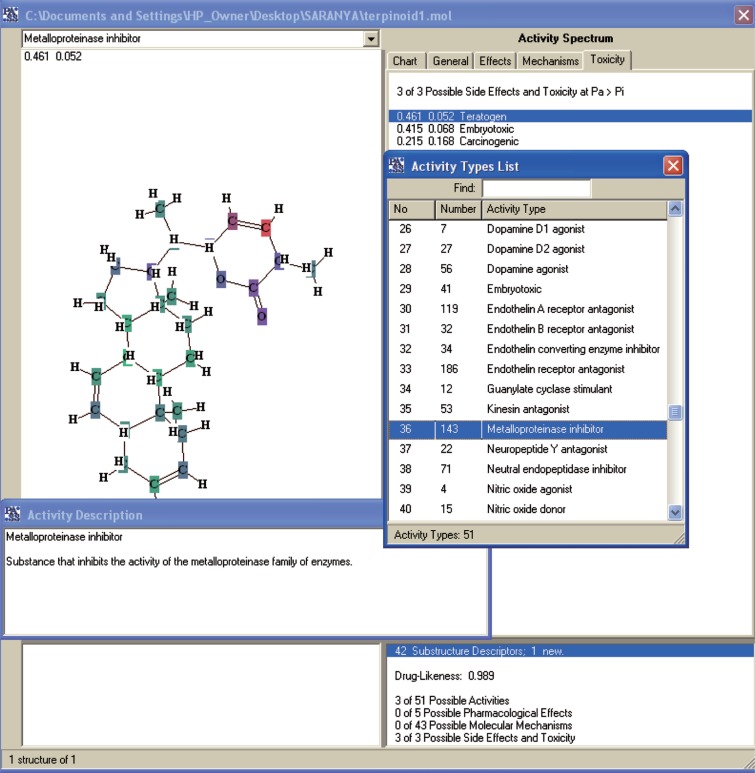
Pass prediction results.

**Figure 5:**
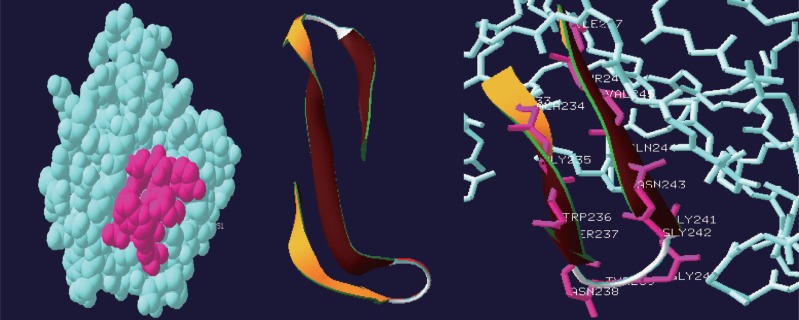
Active site-loop of the target protein (GLY 240, GLY241, GLY242).

**Figure 6:**
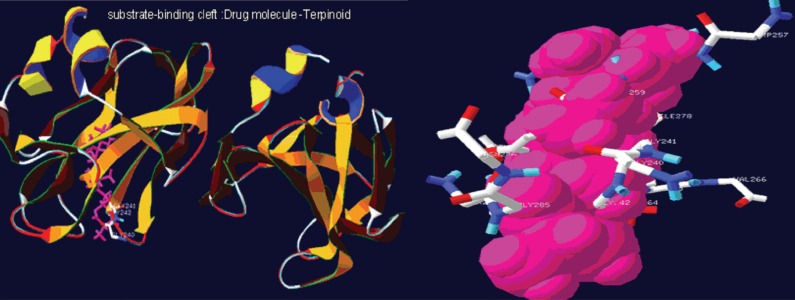
Substrate binding cleft and Protein ligand complex.

**Figure 7:**
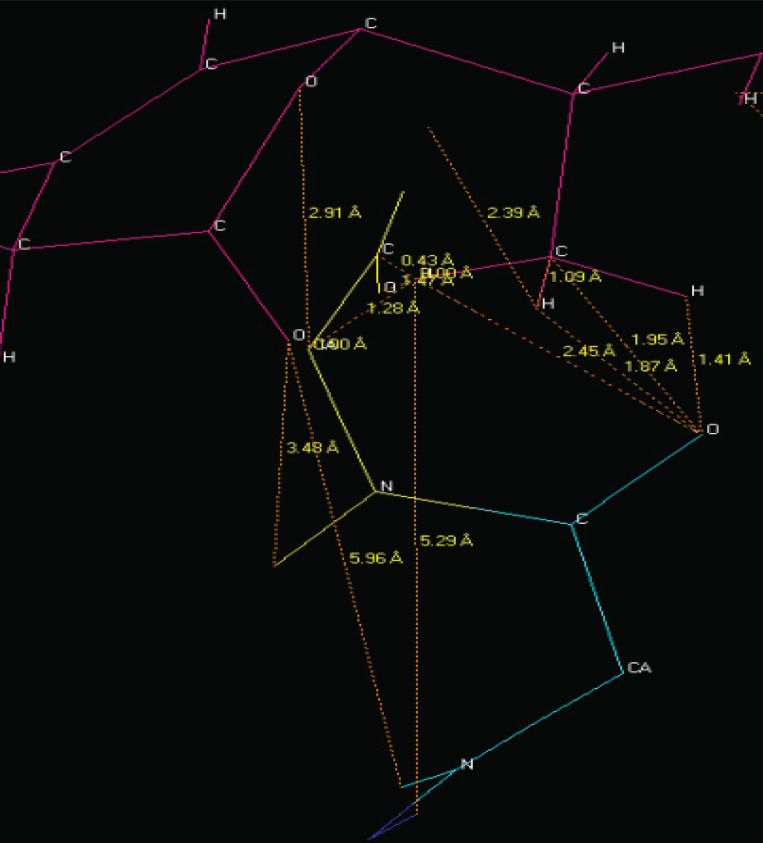
Hydrogen bonds (H-bond) interaction.

**Figure 8:**
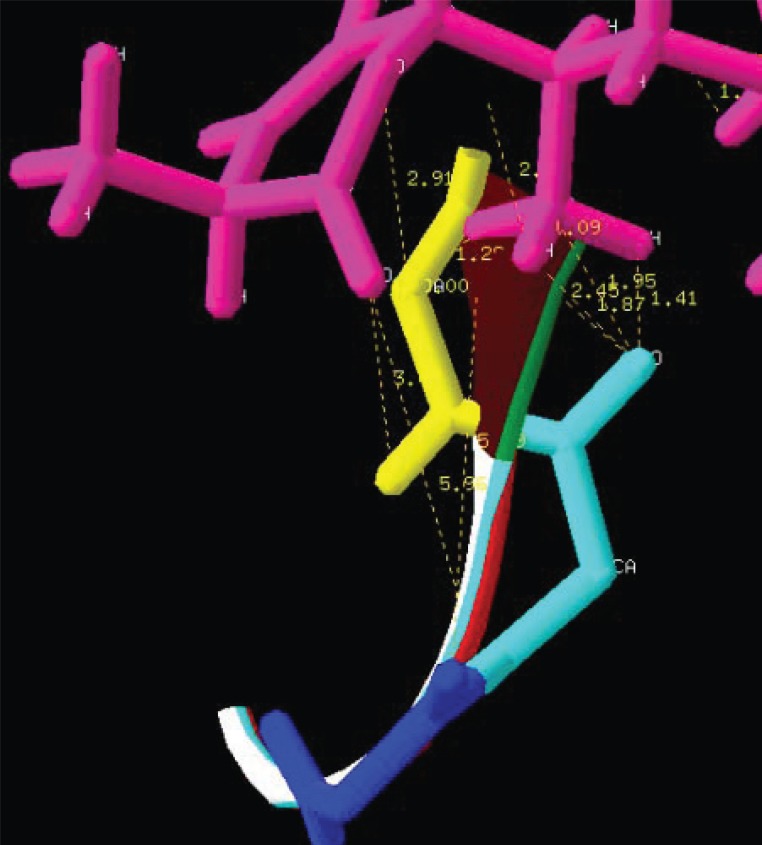
Hydrogen bonds (H-bond) interaction.

The NMR study result of the Terpenoid is given in Fig. 2. Based on the NMR study result of Terpenoid the 3D structure was predicted using the ChemSketch Software and the result is given in Fig. 3 and Table 1 explains the predicted properties of Terpenoid. Fig. 4 is the structural prediction result of Terpenoid using Pass Prediction to Software. This result shows that the compound will act as an inhibitor for the metalloprotease Gly-Gly-Endopeptidase enzyme family. Fig. 5 shows the structure of the active site loop of the target protein which has consecutive Glycine units at the positions 240,241,242. Table 2 showing the docking result. The minimum energy (Emin) value for the protein-ligand complex was calculated. The calculated docking results are given. Fig. 6 depicts the structure binding site of the autolysin protein and the structure of the protein-ligand complex. Fig. 7 & 8 illustrates the computed results of atomic interactions (H-bonds) between the protein and the ligand. Table 3 explains the atoms involved in protein-ligand interaction, calculated Bond length and the positions of amino acid of the active site.

**Table 1:** Predicted Properties of Terpenoid

Calculated properties	Terpanoid

Molecular Formula	C_27_H_38_O_2_
Formula Weight	394.58942
Composition	C (82.18%), H (9.71%), O (8.11%)
Molar Refractivity	117.31 ± 0.3 cm^3^
Molar Volume	376.1 ± 3.0 cm^3^
Parachor	928.8 ± 6.0 cm^3^
Index of Refraction	1.536 ± 0.02
Surface Tension	37.1 ± 3.0 dyne/cm
Density	1.048 ± 0.06 g/cm^3^
Polarizability	46.50 ± 0.5 × 10^-24^ cm^3^
Monoisotopic Mass	394.28718 Da
Nominal Mass	394 Da
Average Mass	394.5894 Da

**Table 2:** The calculated docking results of the minimum energy value for the protein-ligand complex

Docking correlations for 2B0P:MOL2 done in 13 min, 3 sec.
Main pass done in 1 min, 27 sec: 146517 orientations/sec, Evaluated 976004352 orientations
Starting orientation [alpha=0] (Energy=245.92) ranked 424460 in the search
Energy range: E_min_=-209.54 E_max_=-101.11
Clustering found 68 clusters from 500 docking solutions in 0.03 seconds
Etotal -209.5 Eshape -209.5 RMS -1.00

**Table 3:** Calculated results of protein-ligand interaction

LIGAND	PROTEIN	AMINOACID	H-BOND

21^st^ “C”	Carbonyl “O”	GLY 241	1.95A
21^st^ Methyl “H”	Carbonyl “O”	GLY 241	1.41A
21^st^ Methyl “H”	Carbonyl “O”	GLY 241	1.87A
21^st^ Methyl “H”	Carbonyl “O”	GLY 241	2.45A
26^th^ Keto “O”	Amino “H”	GLY 242	3.48A
21^st^ Methyl “H”	C-α	GLY 242	1.28A
Pyranone “O”	C-α	GLY 242	2.91A
21^st^ Methyl “H”	Carbonyl “C”	GLY 242	0.43A
21^st^ Methyl “H”	Carbonyl “O”	GLY 242	1.47A

## DISCUSSION

The active site loop of the target protein containing the 3 consecutive glycine residues does not possess any other functional group other than the amino and the carbonyl group of the peptide bond and they are not involved in the formation of the secondary structures hence these groups are free to have strong atomic interactions with the Terpenoid under study and can easily bind with the loop.

Fig. 6 and Table 3 confirms the presence of free amino and carbonyl group of the protein which is tightly bound with the Terpenoid at the positions 20, 21, 22 and 26 respectively.

Since the protein-ligand complex showed E_min_=-209.54, the novel drug Terpenoid is more compatible with the receptor active site. As the terpenoid can act as a Metalloprotease inhibitor, the activity of the target Autolysin, which also a member of the Metaloprotease-glycine glycine endo peptidase, is inhibited.

Three sorts of hypotheses have been suggested about the mechanism of action of autolysin. First, that the enzymes are essential to make openings in the supposedly continuous network of mucopeptides in the wall (31) so that the new building blocks can be added during growth (29); second, that the enzymes are essential for the remodelling of the wall in making the septum that divides one bacterium into two daughter cells (26, 29); and third, that they are necessary to break the two newly formed bacteria apart from each other (23).

From the literature, the essential oil of *Melaleuca alternifolia* (tea tree) has broad-spectrum antimicrobial activity. The mechanisms of action of tea tree oil and three of its components, 1,8-cineole, terpinen-4-ol, and -terpineol, against *S. aureus* ATCC 9144 were investigated (9). Some antimicrobial agents cause gross membrane damage and provoke whole-cell lysis (12, 30). The failure of TTO or its components to lyse *S. aureus* cells suggests that their primary mechanism of action is not gross cell wall damage. Treatment-induced release of membrane-bound cell wall autolytic enzymes will induce lysis eventually (16), the activation of autolytic enzymes may have been responsible for this effect, the lysis may also have been due to weakening of the cell wall and the subsequent rupture of the cytoplasmic membrane due to osmotic pressure (rather than a specific action on the cytoplasmic membrane) (9).

From the above cited literature, it is evident that the terpenoid under study can inhibit all the above mentioned hypotheses.

Hence we conclude from our docking study that, the novel terpenoid isolated from *E.scaber* possesses antibacterial activity (*S.aureus*) by inhibiting the autolysin.
